# Medical Legislation

**Published:** 1901-09-07

**Authors:** 


### Medical Legislation.

The Parliamentary Session which closed last month was singularly unprolific of measures affecting directly or indirectly the medical profession or the public health. But the ever present necessity of regulating the management of factories, workshops and laundries, according to the needs of the moment, occasioned a measure which goes far to consolidate, but by no means closes, the question.

An adverse vote compelled the Government to grant an extra hour of holiday on Saturday to the workers in textile industries, and without endorsing the often stated view that the fewer hours a person works, the better is his output, we must agree that as short a time as possible in an atmosphere frequently charged with particles of dust is good for the individual. The Pure Beer Bill was a striking example, fortunately now very rare, of an attempt to hasten through a measure by the force of a public scare. There is no doubt that arsenic is often found in beer in quantities large enough to cause symptoms of poisoning, and that the metal may be derived from a variety of commodities used in brewing. Sugar, malt, and the coke used in the malt kilns may all be sources of contamination, but a great deal of work lies before the chemist before any radical working method can be devised to meet and remedy the evil. From a medical standpoint the question has to be decided whether peripheral neuritis is due to arsenic, to alcohol, or to a combination of the two; whether the alcohol derived from the distillation or fermentation of the sugar from grain has a different effect to that produced by the fermentation of the sugar from the grape; whether, in other words, beer, whisky, brandy and wine drinkers are affected differently. All this can only be discovered by a careful analysis of the numbers of people affected in various countries where different alcoholic beverages are consumed. There are, indeed, some who are affected by the violent reaction from old ideas which often attends new discoveries and who are doubtful if peripheral neuritis is caused by alcohol at all and others again who doubt if arsenic is really the poison which is causing the symptoms.

Under these circumstances it seems impossible to draw up any measure which has much chance of being permanent, and it is far better to wait until some definite facts have been discovered before resorting to legislation. One thing is certain, the recent scare has caused every brewery in the kingdom to analyse carefully their ingredients, and we may rest assured that for some time at least beer will not be issued to the public containing poisonous doses of arsenic. Arsenic we must remember is worldwide in its distributions, and the chemical tests for it are so delicate that the merest trace can be detected. We must therefore be prepared to hear that the metal has been discovered in various other articles of food, just as a few years ago we were informed every few days of the number of microbes in every mouthful of bread and butter we consumed.

Little has therefore been accomplished during the last Session, but there are many subjects which are rapidly ripening for legislation. Of these the regulation of midwives is a question of urgent importance. The object is to ensure that the poorer sections of the community shall have the benefit of adequately trained midwives or nurses to attend them during the period of labour and afterwards until the puerperal dangers have passed. It is certain that the fees of trained nurses are beyond the means of many, and it is therefore proposed by the Medical Guild of Manchester that the local authorities should administer a scheme by which registered obstetric nurses should be placed at the disposal of the patients free of charge, and who should be assisted whenever necessary by a qualified practitioner. One could hardly expect a mere nurse, however, to diagnose any abnormal presentation or other unusual feature in the labour until the symptoms have become marked, and thus the difficulties of treatment would be greatly enhanced for the practitioner. In this way the danger to the mother would be greatly increased while infant mortality would probably also rise. It seems, therefore, that according to this scheme the chief requisition must be the early attendance of a qualified practitioner who would assure himself that the labour would in all probability be a normal one, and then leave it in the hands of the nurse who could resort to his services if she found them necessary, and it remains to be seen whether the ratepayers are prepared to provide and pay for the services of a qualified medical practitioner for every parturient woman who says she is unable to pay for such assistance herself. If the public decline to provide the doctor, and the women persist in calling in the midwife, it seems not unreasonable to demand that the latter shall give proper guarantees as to her conduct and her education.

